# Supervised machine learning classification of psychosis biotypes based on brain structure: findings from the Bipolar-Schizophrenia network for intermediate phenotypes (B-SNIP)

**DOI:** 10.1038/s41598-023-38101-0

**Published:** 2023-08-10

**Authors:** Joshua D. Koen, Leslie Lewis, Michael D. Rugg, Brett A. Clementz, Matcheri S. Keshavan, Godfrey D. Pearlson, John A. Sweeney, Carol A. Tamminga, Elena I. Ivleva

**Affiliations:** 1 Center for Vital Longevity, University of Texas at Dallas, Dallas, TX USA; 2 Department of Psychology, University of Notre Dame, Notre Dame, IN 46556 USA; 3 UT Southwestern Medical Center, Dallas, TX USA; 4 University of East Anglia, Norwich, UK; 5 University of Georgia, Athens, GA USA; 6 Harvard Medical School, Beth Israel Deaconess Hospital, Boston, MA USA; 7 Institute of Living, Hartford Hospital, Hartford, CT USA; 8 Yale School of Medicine, New Haven, CT USA; 9 University of Cincinnati, Cincinnati, OH USA

**Keywords:** Machine learning, Cognitive neuroscience, Human behaviour

## Abstract

Traditional diagnostic formulations of psychotic disorders have low correspondence with underlying disease neurobiology. This has led to a growing interest in using brain-based biomarkers to capture biologically-informed psychosis constructs. Building upon our prior work on the B-SNIP Psychosis Biotypes, we aimed to examine whether structural MRI (an independent biomarker not used in the Biotype development) can effectively classify the Biotypes. Whole brain voxel-wise grey matter density (GMD) maps from T1-weighted images were used to train and test (using repeated randomized train/test splits) binary L2-penalized logistic regression models to discriminate psychosis cases (n = 557) from healthy controls (CON, n = 251). A total of six models were evaluated across two psychosis categorization schemes: (i) three Biotypes (B1, B2, B3) and (ii) three DSM diagnoses (schizophrenia (SZ), schizoaffective (SAD) and bipolar (BD) disorders). Above-chance classification accuracies were observed in all Biotype (B1 = 0.70, B2 = 0.65, and B3 = 0.56) and diagnosis (SZ = 0.64, SAD = 0.64, and BD = 0.59) models. However, the only model that showed evidence of specificity was B1, i.e., the model was able to discriminate B1 vs. CON and did not misclassify other psychosis cases (B2 or B3) as B1 at rates above nominal chance. The GMD-based classifier evidence for B1 showed a negative association with an estimate of premorbid general intellectual ability, regardless of group membership, i.e. psychosis or CON. Our findings indicate that, complimentary to clinical diagnoses, the B-SNIP Psychosis Biotypes may offer a promising approach to capture specific aspects of psychosis neurobiology.

## Introduction

Current diagnostic approaches in psychiatry are based almost exclusively on phenomenological observations rather than biological verification. Unlike other medical fields, in psychiatry objective biomarker measures are rarely used to support clinical decision-making. Growing evidence indicates that the existing diagnostic formulations show poor correspondence to emerging biomarker-based constructs^1,2^. The absence of biologically-informed disease entities—e.g. for psychotic disorders—or actionable biomarkers within the global disease constructs hampers progress in understanding disease mechanisms and treatment development.

Recent strategies using underlying neurobiology have challenged diagnostic boundaries of psychoses. Instead of searching for biomarkers for ‘schizophrenia’ or ‘bipolar disorder’, data-driven approaches using broad biomarker panels have been applied to re-categorize psychosis cases into subgroups with more cohesive neurobiological profiles^3,4^. Using this approach, the Bipolar-Schizophrenia Network for Intermediate Phenotypes (B-SNIP) recently developed biomarker-based psychosis constructs—Biotypes—that capture biologically-distinctive groups of psychosis cases^3^. Based on a multistep multivariate analyses using cognition, EEG, and oculomotor measures, three distinctive Biotypes emerged: Biotype1 (B1), characterized by poor cognitive and low sensorimotor function; Biotype2 (B2), with moderately impaired cognition and exaggerated sensorimotor reactivity; and Biotype3 (B3), with near normal cognitive and sensorimotor functions^3^. Notably, the conventional diagnoses [schizophrenia (SZ), schizoaffective disorder (SAD), psychotic bipolar I disorder (BD)] mapped poorly onto the Biotypes, with all three diagnoses distributed across all Biotypes. Analysis of whole brain voxel-wise morphometry—an “external validator” not used in Biotype development—demonstrated a step-wise pattern of gray matter density (GMD) reductions across the Biotypes: in B1, extensive and diffusely distributed GMD loss, with the largest effects in frontal, anterior/middle cingulate, and temporal regions; in B2, intermediate in magnitude and more localized reductions, with the largest effects in insula and fronto-temporal regions; and in B3, modest GMD reductions primarily localized to anterior limbic regions^5^. In the same sample segregated by conventional diagnoses, we observed highly similar (and diffusely distributed) GMD reductions in SZ and SAD, and modest, primarily frontal reductions in BD. Biotypes showed better between-group discrimination based on GMD features and were a stronger predictor of GMD alterations than the diagnoses. Additionally, distinct patterns of resting state connectivity^6^ emerged across the Biotypes.

There has been a growing interest in using machine learning approaches to disentangle the heterogeneity of mental illness. A critical question is whether there are biological measures—especially those commonly used in clinical practice, such as structural MRI—that can accurately predict different psychosis groups according to conventional diagnoses or, of importance here, to a Biotypes classification scheme. Prior research has primarily focused on using structural MRI measures (e.g., GMD/volume, cortical thickness) to train classification algorithms in order to discriminate psychosis groups (mainly SZ and BD) from healthy controls (CON). Previous reports have demonstrated that gray matter-focused features can discriminate SZ vs. CON with high levels of accuracy (0.66–0.89)^7–12^, including in multi-site datasets^13,14^ (for related findings from “fusion” approaches, see Refs.^15–19^). Studies in BD have found somewhat lower classification accuracies for BD vs. CON (0.61–0.78)^20–22^ relative to those for SZ. Few studies have attempted to discriminate SZ vs. BD in a single analysis, and yielded modest classification accuracies (e.g., 0.66 in Ref.^23^). The limited ability to differentiate between SZ and BD is likely due to the significant biological heterogeneity of the disorders, as previously demonstrated in our Refs.^3,5^ and others’ work^24–30^. Recently, Mothi et al.^31^ investigated the utility of unsupervised machine learning for delineating psychosis subgroups in the B-SNIP sample. Integrating symptom-based ratings and biomarker data, they identified three distinct subgroups (called “G1, G2 and G3”, different from the Biotypes). Subsequent analysis of external validators showed that the subgroups differed significantly in cortical thickness, oculomotor and general and social functioning measures, with G1 showing the greatest, and G3, the least impairments. Similar to the B-SNIP Biotypes, the psychosis subgroups identified in this study showed only limited correspondence with conventional diagnoses^31^.

Building on our prior work demonstrating distinct VBM-based GMD alterations among the B-SNIP Biotypes^3,5^, we used a supervised machine learning classification approach to determine whether GMD characteristics can reliably discriminate between psychosis groups—categorized either according to Biotype or conventional diagnosis—and healthy individuals. We hypothesized that a GMD-based classifier would show more specificity for biologically-defined Biotypes relative to symptom-based diagnoses. In addition, we explored whether GMD-based classifier output for Biotypes is associated with clinical and other biomarker measures. This exploratory analysis aimed to examine if the GMD-based classifier captures dimensions of psychosis that fall along a continuum that is present in both psychosis cases and CON (i.e., independent of group membership).

## Methods

### Study sample

Voxel-wise GMD metrics from the Voxel-Based Morphometry pipeline^32,33^ were extracted in 808 subjects [557 psychosis, 251 CON] initially categorized according to Biotype, and then, by DSM diagnoses (for demographic and clinical data, see Table 1 and Supplemental Table S1).

**Table 1:** Socio-demographic and clinical characteristics of the study sample by Biotype and Conventional Diagnosis.

Sample characteristics by Biotype						
	B1 (n = 150)	B2 (n = 185)	B3 (n = 222)	CON (n = 251)	Test statistic	*p* value
Socio-demographic characteristics						
Age, years; mean (SD)	35.3 (13.1)	35.4 (11.9)	35.3 (12.6)	36.9 (12.1)	*F*(3, 804) = 0.93	0.42
Sex/Male; n (%)	80 (53.3)	87 (47.0)	116 (52.3)	109 (43.4)	χ^2^ (3) = 5.38	0.15
Handedness; n (%)						
Right-handed	127 (84.7)	163 (88.1)	188 (84.7)	217 (86.5)	χ^2^ (6) = 4.84	0.56
Left-handed	17 (11.3)	18 (9.7)	28 (12.6)	30 (12.0)		
Ambidextrous	5 (3.3)	2 (1.1)	4 (1.8)	2 (0.8)		
Ethnicity/Hispanic; n (%)	18 (12.0)	20 (9.7)	12 (5.4)	26 (10.4)	χ^2^ (3) = 6.0	0.11
Race; n (%)						
Caucasian	58 (38.7)	114 (61.6)	146 (65.8)	166 (66.1)	χ^2^ (6) = 43.2	< 0.001^a^
African-American	81 (54.0)	57 (30.8)	60 (27.0)	62 (24.7)		
Other	11 (7.3)	14 (7.6)	16 (7.2)	23 (9.2)		
Education, years; mean (SD)	12.4 (2.0)	13.1 (2.3)	14.1 (2.3)	15.1 (2.5)	*F*(3, 803) = 52.84	< 0.001^b^
Clinical characteristics; mean (SD)						
Age of illness onset, years	20.5 (7.4)	20.8 (8.9)	20.3 (8.6)	–	*F*(2, 533) = 0.14	0.87
Age of first hospitalization, years	22.2 (8.2)	23.2 (8.4)	23.3 (8.6)	–	*F*(2, 487) = 0.72	0.49
Number of lifetime hospitalizations	5.6 (6.9)	5.8 (6.6)	5.0 (8.3)	–	*F*(2, 450) = 1.89	0.15
PANSS						
Total	64.7 (17.6)	64.0 (17.1)	60.3 (16.2)	–	*F*(2, 544) = 3.85	< 0.02^c^
Positive subscale	16.6 (5.8)	16.1 (5.9)	15.4 (5.2)	–	*F*(2, 545) = 2.34	0.10
Negative subscale	16.3 (5.7)	15.3 (5.4)	13.9 (5.2)	–	*F*(2, 545) = 9.85	< 0.001
General psychopathology subscale	31.8 (9.1)	32.6 (9.0)	31.1 (8.6)	–	*F*(2, 546) = 1.53	0.22
YMRS	5.7 (5.7)	6.7 (6.6)	6.1 (6.2)	–	*F*(2, 541) = 0.85	0.43
MADRS	10.0 (9.7)	11.1 (9.0)	10.5 (9.3)	–	*F*(2, 541) = 0.61	0.55
GAF	48.9 (11.8)	53.1 (13.9)	55.8 (13.8)	86.6 (6.5)	*F*(3, 795) = 482.0	< 0.001^d^
BACS	− 2.6 (0.9)	− 1.9 (0.9)	− 0.2 (0.8)	− 0.02 (1.2)	*F*(3, 777) = 315.5	< 0.001^e^
WRAT-4	89.6 (13.1)	95.3 (13.6)	105.9 (14.4)	103.5 (13.8)	*F*(3, 796) = 54.08	< 0.001f.
SFS	117.4 (23.6)	124.4 (23.7)	132.0 (24.9)	157.7 (16.4)	*F*(3, 621) = 100.3	< 0.001^ g^
Concomitant medications; n (%)						
Off psychotropic medications	2 (1.3)	13 (7.0)	18 (8.1)	240 (95.6)	–	–
Antipsychotics	140 (93.3)	159 (85.9)	170 (76.6)	0 (0.0)	–	–
CPZ equivalents	542.5(436.5)	502.7(469.7)	392.5(312.8)	–	*F*(2, 352) = 2.29	0.01^ h^
Mood stabilizers						
Lithium	14 (9.3)	25 (13.5)	36 (16.2)	0 (0.0)	–	–
Other	51 (34.0)	59 (31.9)	73 (32.9)	0 (0.0)	–	–
Antidepressants	57 (38.0)	83 (44.9)	102 (45.9)	3 (1.2)	–	–
Anxiolytics/Hypnotics	37 (24.7)	53 (28.6)	59 (26.6)	6 (2.4)	–	–
Anticholinergics	35 (23.3)	15 (8.1)	26 (11.7)	0 (0.0)	–	–
Stimulants	10 (6.7)	9 (4.9)	19 (8.6)	2 (0.8)	–	–
Other	2 (1.3)	6 (3.2)	6 (2.7)	0 (0.0)	–	–
Combined medications	125 (83.3)	141 (76.2)	160 (72.1)	3 (1.2)	–	–

Sample characteristics by DSM-IV diagnosis						
	SZ (n = 242)	SAD (n = 138)	BD (n = 177)	CON (n = 251)	Test statistic	*p* value
Socio-demographic characteristics						
Age, years; Mean (SD)	34.4 (12.3)	36.0 (11.9)	36.1 (13.1)	36.9 (12.1)	*F*(3, 804) = 1.70	0.16
Sex/Male; n (%)	165 (68.2)	58 (42.0)	60 (33.9)	109 (43.4)	χ^2^ (3) = 57.27	< 0.001^i^
Handedness; n (%)						
Right-handed	208 (86.0)	120 (87.0)	150 (84.7)	217 (86.5)	χ^2^ (6) = 10.03	0.12
Left-handed	26 (10.7)	12 (8.7)	25 (14.1)	30 (12.0)		
Ambidextrous	4 (1.7)	7 (4.3)	1 (0.6)	2 (0.8)		
Ethnicity/Hispanic; n (%)	21 (8.7)	16 (11.6)	13 (7.3)	26 (10.4)	χ^2^ (3) = 2.06	0.56
Race; n (%)						
Caucasian	112 (46.3)	75 (54.3)	131 (74.0)	166 (66.1)	χ^2^ (6) = 44.28	< 0.001^j^
African-American	109 (45.0)	54 (39.1)	35 (19.8)	62 (24.7)		
Other	21 (8.7)	9 (6.5)	11 (6.2)	23 (9.2)		
Education, years; mean (SD)	12.8 (2.2)	13.1 (2.2)	14.2 (2.4)	15.1 (2.5)	*F*(3, 803) = 47.8	< 0.001^ k^
Clinical characteristics; mean (SD)						
Age of illness onset, years	21.3 (7.5)	20.2 (9.1)	19.8 (8.8)	–	*F*(2, 533) = 1.71	0.18
Age of first hospitalization, years	22.6 (7.0)	22.6 (8.7)	23.8 (9.8)	–	*F*(2, 487) = 0.95	0.39
Number of lifetime hospitalizations	5.6 (8.2)	6.2 (6.1)	5.5 (7.2)	–	*F*(2, 450) = 0.29	0.75
PANSS						
Total	65.5 (17.2)	68.5 (16.1)	54.2 (13.9)	–	*F*(2, 544) = 37.3	< 0.001^ l^
Positive subscale	16.7 (5.7)	18.2 (5.2)	13.1 (4.6)	–	*F*(2, 545) = 41.9	< 0.001
Negative subscale	16.7 (6.1)	15.6 (4.7)	12.2 (3.9)	–	*F*(2, 545) = 40.0	< 0.001
General psychopathology subscale	32.1 (8.8)	34.7 (8.9)	28.9 (8.2)	–	*F*(2, 546) = 17.7	< 0.001
YMRS	5.7 (5.7)	7.3 (6.3)	5.9 (6.7)	–	*F*(2, 541) = 3.0	0.051
MADRS	8.5 (8.1)	14.6 (10.4)	10.3 (8.9)	–	*F*(2, 541) = 20.3	< 0.001^ m^
GAF	49.5 (12.6)	48.8 (11.7)	61.3 (12.5)	86.6 (6.5)	*F*(3, 795) = 586.1	< 0.001^n^
BACS	− 1.8 (1.3)	− 1.5 (1.3)	− 0.9 (1.3)	− 0.02 (1.2)	*F*(3, 777) = 83.7	< 0.001^o^
WRAT-4	95.4 (16.0)	96.4 (14.8)	102.9 (13.7)	103.5 (13.8)	*F*(3, 796) = 18.0	< 0.001^p^
SFS	122.2 (24.3)	119.0 (25.4)	135.4 (22.1)	157.7 (16.4)	*F*(3, 621) = 105.6	< 0.001^q^
Concomitant medications; n (%)						
Off psychotropic medications	13 (5.4)	7 (5.1)	13 (7.3)	240 (95.6)	–	–
Antipsychotics	218 (90.1)	122 (88.4)	129 (72.9)	0 (0.0)	–	–
CPZ equivalents	527.0(407.1)	536.8(463.9)	328.0(327.8)	–	*F*(2, 352) = 9.22	< 0.001^r^
Mood stabilizers						
Lithium	14 (5.8)	16 (11.6)	45 (25.4)	0 (0.0)	–	–
Other	41 (16.9)	62 (44.9)	80 (45.2)	0 (0.0)	–	–
Antidepressants	87 (36.0)	78 (56.5)	77 (43.5)	3 (1.2)	–	–
Anxiolytics/Hypnotics	55 (22.7)	38 (27.5)	56 (31.6)	6 (2.4)	–	–
Anticholinergics	40 (16.5)	20 (14.5)	16 (9.0)	0 (0.0)	–	–
Stimulants	13 (5.4)	7 (5.1)	18 (10.2)	2 (0.8)	–	–
Other	5 (2.1)	5 (3.6)	4 (2.2)	0 (0.0)	–	–
Combined medications	164 (67.8)	121 (87.7)	141 (79.7)	3 (1.2)	–	–

*B1* Biotype 1, *B2* Biotype 2, *B3* Biotype 3, *CON* healthy controls, *SZ* schizophrenia, *SAD* schizoaffective disorder, *BD* psychotic bipolar I disorder, *SD* standard deviation, *PANSS* the Positive and Negative Syndrome Scale, *YMRS* the Young Mania Rating Scale, *MADRS* the Montgomery–Asberg Depression Rating Scale, *GAF* the Global Assessment of Functioning, *BACS* Brief Assessment of Cognition in Schizophrenia, *WRAT-4* premorbid general intelligence estimate based on Wide Range Achievement Test-4 (reading subtest), *SFS* the Birchwood Social Functioning Scale, *CPZ equivalents* daily antipsychotic dose chlorpromazine equivalents.

A one-way analysis of variance with a subsequent post hoc Tukey Honestly Significant Difference test and Yates corrected chi-square test were used, as appropriate, for demographic and clinical variables; only statistically significant (*p* < 0.05) between-group differences are reported.

Biotype constructs: ^a^Race: B1 had a higher proportion of African-Americans than Caucasians relative to B2 [χ^2^ (1) = 18.31, *p* < 0.001], B3 [χ^2^ (1) = 27.98, *p* < 0.001] and CON [χ^2^ (1) = 33.78, *p* < 0.001]. ^b^Education: B1 had fewer years of education than B2 (*p* = 0.014), B3 (*p* < 0.001) and CON (*p* < 0.001). B2 had lower education than B3 (*p* < 0.001) and CON (*p* < 0.001). B3 had fewer years of education than CON (*p* < 0.001). ^c^PANSS: Total score: B1 had higher score than B3 (*p* = 0.04). Negative symptoms subscale: B1 (*p* < 0.001) and B2 (*p* = 0.02) had higher scores than B3. ^d^GAF: All Biotype groups had lower scores than CON (all *p* < 0.001). B1 had lower scores than B2 (*p* = 0.007) and B3 (*p* < 0.001). ^e^BACS: B1 had lower score than B2, B3 and CON (all *p* < 0.001). B2 had lower score than B3 and CON (both *p* < 0.001). ^f^WRAT-4 IQ: B1 had lower score than B2 (*p* = 0.001), B3 (*p* < 0.001) and CON (*p* < 0.001). B2 had lower score than B3 (*p* < 0.001) and CON (*p* < 0.001). ^g^SFS: B1 had lower score than B2 (*p* = 0.049), B3 (*p* < 0.001) and CON (*p* < 0.001). B2 had lower score than B3 (*p* < 0.01) and CON (*p* < 0.001). B3 had lower score than CON (*p* < 0.001). ^h^Daily antipsychotic dose CPZ equivalents by Biotype: B1were treated with higher daily doses of antipsychotic medications than B3 (*p* = 0.017).

Conventional diagnoses: ^i^Sex: There was a higher proportion of males among SZ compared to SAD [χ^2^ (1) = 23.73, *p* < 0.001], BD [χ^2^ (1) = 46.96, *p* < 0.001] and CON [χ^2^ (1) = 29.59, *p* < 0.001]. ^j^Race: SZ had a higher proportion of African-Americans than Caucasians relative to BD [χ^2^ (1) = 31.15, *p* < *0*.001] an CON [χ^2^ (1) = 22.37, *p* < 0.001]. SAD had a higher proportion of African-Americans than Caucasians compared to BD [χ^2^ (1) = 13.90, *p* < 0.001] and CON [χ^2^ (1) = 7.43, *p* = 0.006]. ^k^Education: SZ had fewer years of education than BD (*p* < 0.001) and CON (*p* < 0.001). SAD had lower education than BD (*p* < 0.001) and CON (*p* < 0.001). BD had lower education than CON (*p* < 0.001). ^l^PANSS: Total score: SZ and SAD had higher scores than BD (both *p* < 0.001). PANSS positive subscale: SAD had higher scores than SZ (*p* = 0.02) and BD (*p* < 0.001); SZ had higher scores than BD (*p* < 0.001). PANSS negative subscale: SZ and SAD had higher scores than BD (both *p* < 0.001). PANSS general subscale: SAD had higher scores than SZ (*p* = 0.01) and BD (*p* < 0.001); SZ had higher scores than BD (*p* < 0.001). ^m^MADRS: SAD had higher scores than SZ and BD (both *p* < 0.001). ^n^GAF: All psychosis groups scored lower than CON (all *p* < 0.001). SZ had lower scores than BD (*p* < 0.001). SAD had lower scores than BD (*p* < 0.001). ^o^BACS: SZ had a lower score than BD and CON (both *p* < 0.001). SAD had a lower score than BD and CON (both *p* < 0.001). BD had a lower score than CON (*p* < 0.001). ^p^WRAT-4 IQ: SZ had a lower score than BD (*p* < 0.001) and CON (*p* < 0.001). SAD had a lower score than BD (*p* = 0.002) and CON (*p* < 0.001). ^q^SFS: All psychosis groups scored lower than CON (all *p* < 0.001). SZ had lower scores than BD (*p* < 0.001). SAD had lower scores than BD (*p* < 0.001). ^r^Daily antipsychotic dose CPZ equivalents: Both SZ and SAD were treated with higher daily doses of antipsychotic medications compared to BD (both *p* < 0.001).

The B-SNIP study’s logistics and overall sample characteristics are described elsewhere^34^. Psychosis subjects were stable, medicated outpatients. CON subjects had no personal history of psychotic or recurrent mood disorders and no family history of schizophrenia/bipolar spectrum disorders in first- or second-degree relatives. Psychiatric diagnoses (and absence thereof in CON) were established via formal diagnostic consensus conferences including a review of the Structured Clinical Interview for DSM-IV-TR Diagnosis (SCID-I/P)^35^ and all available clinical information. The study was approved by Institutional Reviews Boards at all B-SNIP data collection sites: (1) University of Texas Southwestern Medical Center, (2) Olin Neuropsychiatry Research Institute, Hartford Hospital, Yale School of Medicine, (3) Maryland Psychiatric Research Center, University of Maryland School of Medicine, (4) University of Illinois at Chicago, (5) Wayne State University, School of Medicine, (6) Harvard University Medical School. All subjects provided written informed consent after the study procedures had been fully explained, and all study procedures were performed in accordance with relevant guidelines and regulations.

### Gray matter density parameters extraction for machine learning analyses

T1-weighted structural images were acquired on 3 T MRI scanners at 5 B-SNIP sites. Magnetization Prepared Rapid Gradient Echo (MPRAGE) or Inversion Recovery-Prepared Spoiled Gradient-Echo (IR-SPGR) sequences, as appropriate for the scanner brands, were used; image parameters were consistent with the Alzheimer's Disease Neuroimaging Initiative (ADNI1) protocol (http://adni.loni.usc.edu/methods/documents/mri-protocols/). Images were preprocessed and analyzed using the optimized Voxel-Based Morphometry^32^ toolbox (VBM8) for Statistical Parametric Mapping (SPM8) (http://www.fil.ion.ucl.ac.uk/spm/software/spm8). The analysis pipeline incorporated the Diffeomorphic Anatomical Registration Through Exponentiated Lie Algebra (DARTEL), a high-dimensional nonlinear inter-subject registration tool^33,36^. The MRI parameters, quality control procedures, and pipeline are detailed in Supplemental Methods.

GMDs were extracted from the segmented and modulated gray matter images (smoothed at 8 mm FWHM) within a gray matter mask. We adopted similar procedures to prior work (e.g. Ref.^37^) to restrict the classification analyses to grey matter voxels. The mask included voxels that met two criteria: values > 0.40 and < 0.60 in the grey and white matter tissue probability maps, respectively. These thresholds were selected based on visual inspection by the first author to minimize including an excessive amount of white matter voxels in the mask and to minimize partial volume effects. Although visual inspection is a common and generally desirable step in imaging analysis as it allows to “screen-out” gross artifacts and other image irregularities, it can be limited because the decision processes can be difficult to replicate. We have included the specific mask in the Open Science Framework repository for this project to support reproducibility of the analyses reported here (https://osf.io/9ra6j/?view_only=02b0bd7639c64bddbb6ecc4903c1e5d7). No further feature reduction or selection steps were included in the analysis pipeline. Thus, all 371,243 features were used in the machine learning analyses.

### Clinical and biomarker measures associated with machine learning classifier output

We further explored associations between GMD-based classifier performance (specifically, the B1 classification model, the only model that demonstrated ‘specificity’, see “Model training and testing” and “Gray matter density-based classifier performance across the biotypes”) and several clinical and biomarker measures. The variables of interests for these analyses were chosen based on two rationales. First, these data were available on both psychosis and CON subjects which was necessary for testing “brain-behavior” associations dimensionally (i.e. across both psychosis and CON individuals regardless of their group membership). Second, the selected clinical and biomarker variables were not used in the original Biotype development^3^. This allowed us to avoid potential “circularity” in the association analyses. In total, six clinical and biomarker measures that satisfied both rationales were selected for the association analyses: an estimate of premorbid general intellectual ability [the Wide Range Achievement Test-4, Word Reading subtest (WRAT-4)], oculomotor function [the Smooth Pursuit Eye Movement (SPEM) task], EEG [intrinsic EEG activity (IEA) derived from inter-stimulus intervals during an auditory paired stimuli task^38^], and general and social functioning [DSM-IV Axis V: Global Assessment of Functioning Scale (GAF), Birchwood Social Functioning Scale (SFS)^34^]. The SPEM and IEA data were reduced via a principal component analysis (PCA), yielding two PCA components for SPEM and one PCA component for IEA. Proportions of subjects with missing data across the 6 variables of interest, comparable across the Biotype and CON groups, are shown in Supplemental Table S2.

### Machine learning analyses

#### Model training and testing

Machine learning analyses examined if patterns of GMDs can reliably classify psychosis cases organized by Biotype or diagnosis. We used a repeated train/test split approach with 1000 iterations. For each iteration, a randomly selected a subset of the data was used to train the classification model, and the held-out data was used to test the performance of the classification model. All classification models were based on L2-normed logistic regression models (penalty = 1) using the *liblinear* package^39^ implemented in the Princeton MVPA toolbox (https://github.com/princetonuniversity/princeton-mvpa-toolbox).

The following methods apply to each iteration of the repeated train/test split approach. A random sample of 88 cases from each Biotype (B1, B2, B3) and from CON was used for model training. This training set size was selected to ensure a minimum of 50 test cases in each group. The DSM categorization was not considered for case selection for the Biotype groups. The remaining cases were used as the test set (B1, n = 62; B2, n = 97; B3, n = 134; CON, n = 163). Three binary classification models were trained that discriminated one of the three Biotype groups vs. CON. The same n = 88 CON training sample was used for the three classification models in each iteration. The trained models were then applied to (i.e., tested on) every case in the held-out test groups. Classifier accuracy was computed using a balanced accuracy metric (i.e., unweighted average of each groups classification accuracy, or the average of the sensitivity and specific of the classifier) given the unequal number of cases in number of cases between two classes in the test data. A model’s classification accuracy was determined to be significant if the 99.17% confidence interval (CI) for overall classifier accuracy (i.e., aggregate classification accuracy across both groups in the model) across the 1,000 repeated train/test iterations did not encompass the nominal chance value of 50%. We used a 99.17% CI as a conservative approach to control for multiple comparisons (three) in the overall classification accuracies within each approach, i.e., Biotype or diagnosis.

Importantly, a model for each Biotype (e.g., B1) was also tested on the other two Biotype groups (e.g., B2 and B3), allowing assessment of the ‘specificity’ of each model. The idea behind this approach is that if a given model (e.g., B1) classifies the designated Biotype group above nominal chance but fails to do so for the other two Biotypes, then the model is likely identifying GMD features that are specific to a particular Biotype group. If, however, a model classifies, or labels, the other Biotype groups at rates exceeded nominal chance, then the model is likely identifying non-specific GMD features indicative of psychosis as a whole.

The above methods were also employed to examine classification of the three diagnostic groups [SZ, SAD, BD]. Case selection for each iteration was not stratified with respect to the Biotype membership. The training set size was identical to that described above, and the remaining cases [SZ, n = 154; SAD, n = 50; BD, n = 89; CON, n = 163] were held out from model training to allow a test of classifier accuracy. Each diagnosis model (e.g., SZ vs. CON) was applied to all cases in the test set for the other diagnostic groups (e.g., SAD and BD) to test model specificity.

The classification accuracy data for all models were analyzed in R^40^, and the raincloud figures used to visualize the data were created using ggplot2^41^. The feature importance weights for each voxel in each model were used to create feature importance maps following a similar procedure as the classification accuracy measures. The procedures specific to this are described in the Supplemental Methods, and a brief description of these data are provided in “Feature weights for the classification models” below.

#### Associations between brain structure-based classifiers and other biomarker and clinical measures

We carried out exploratory analyses examining the relationship between GMD-based classifier output and additional biomarker and clinical measures (described in “Clinical and biomarker measures associated with machine learning classifier output”). These exploratory analyses were restricted to classifier output from B1 classification model because it was the only model that demonstrated ‘specificity’ (see “Model training and testing” and “Gray matter density-based classifier performance across the biotypes”). We conducted a series of regression analyses that predicted clinical and biomarker measures given classifier output (i.e., prediction probability) from the B1 model. This analysis was conducted on the entire test sample from each iteration of repeated train/test splits of the data. Using the cases across all four groups allowed us to test for an interaction between GMD-classifier evidence and group membership, and the clinical and biomarker measures. The rationale for this analysis is rooted in the idea that the GMD-based classifier model may provide a sensitive measure that can reliably predict an individual’s clinical and/or neurobiological profile regardless of group membership. Finding a group-invariant relationship would suggest that this GMD-based classifier approach might capture important biomarker and clinical characteristics that span a “disease/CON” dimension, and that are not characteristic of only a single psychosis group.

Each multiple regression model included the biomarker or clinical measure as the outcome variable and a total of 7 predictor variables: classifier output from the B1 model, three dummy-coded group variables (one for each Biotype group; CONs served as the reference group), and three variables representing the interaction between each dummy-coded group variable and the B1 model evidence. Classifier evidence from the B1 model takes on values between 0 and 1, with higher values indicating increased classifier evidence that a data point showed a pattern of GMD characteristic of B1. B1 classifier evidence was mean centered before creating the interaction terms to reduce multi-collinearity between the predictor variables. The interaction terms allowed us to test if group membership moderates the association between GMD-classifier output and the examined outcome variables. Given the exploratory nature of these analyses, the model term for B1 classifier evidence (and all other regression model terms) was deemed significant if the 95% interval of unstandardized *b* values obtained across the 1,000 iterations of the repeated train/test splits did not include 0.

## Results

### Gray matter density-based classifier performance across the biotypes

**Figure 1:**
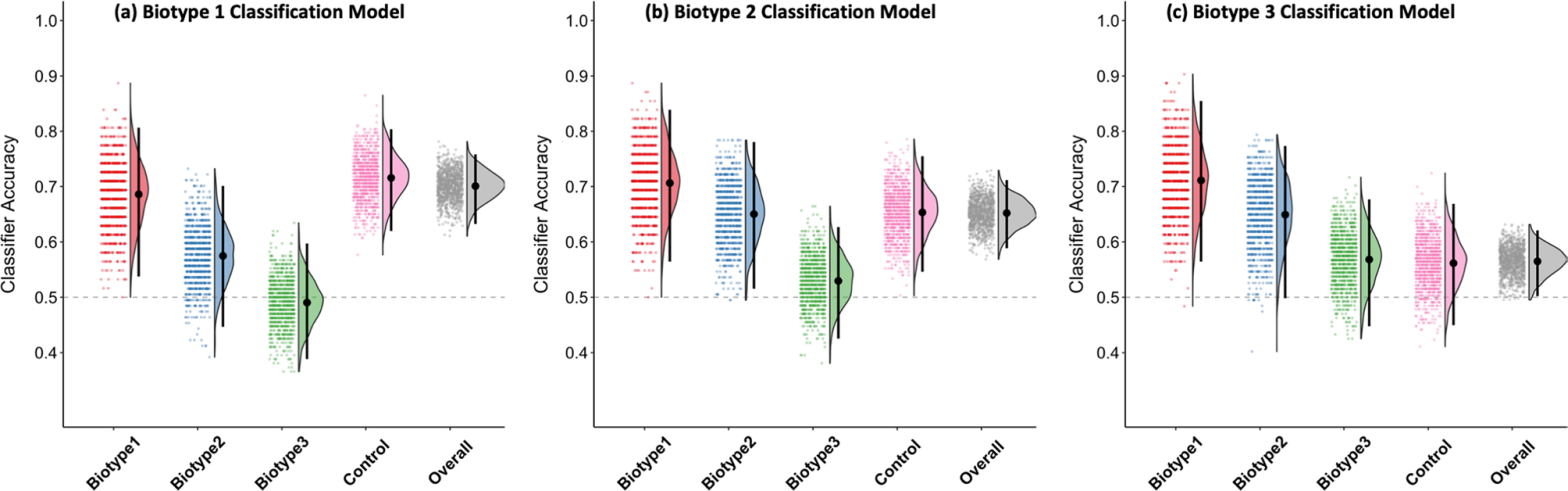
Raincloud plots from the analysis of the GMD classifiers for the (**a**) Biotype group 1 (B1) versus Control (CON), (**b**) Biotype group 2 (B2) versus CON, and (**c**) Biotype group 3 (B3) versus CON. In each panel, the dots represent balanced classifier accuracy for each of the 1000 iterations, the density plot shows the distribution of accuracy values across iterations, and the black dot and line reflects the mean and 99.17% interval of the accuracy values, respectively. Overall accuracy reflects on the two groups in the model (e.g., overall accuracy for the B1 model is the combined accuracy of B1 and CON cases). The other columns of the figure reflect accuracy for individual groups. Note that accuracy for groups not included in the training model (e.g., B2 and B3 for the B1 model), the ‘accuracy’ value reflects the rate of classifier guesses for being in the psychosis group (B1).

The results for the three Biotypes classification models are shown in Fig. 1 and Supplemental Table S3. For the model comparing B1 and CON, overall model classification accuracy was significantly above chance. Classification accuracies were also significantly above chance for both the B1 and CON. Importantly, the model did not classify either B2 or B3 cases as belonging to the B1 group at rates above chance. We interpret this pattern of results as evidence for specificity in discriminating between B1 vs. CON based on GMD features.

For the model comparing B2 and CON, the overall model classification accuracy was significantly above chance. Classification accuracies were also significantly above chance for both B2 and CON. However, this model did not demonstrate specificity: although B3 cases were not misclassified as B2 at above chance levels, B1 cases were misclassified as B2 significantly above chance. Thus, it appears that classification accuracy of the B2 model was driven by GMD features common to both B1 and B2, relative to CON, and not features specific to B2.

Lastly, overall classification accuracy for the B3 vs. CON model was significantly above chance. However, neither the separate classification accuracies for B3 nor for CON exceeded chance levels. Moreover, the model did not show specificity as B1 cases were misclassified as belonging to the B3 group at rates greater than nominal chance. B2 cases were not misclassified as B3 above nominal chance. This pattern of results suggests that classification performance of the B3 model was driven by GMD characteristics common to both the Biotypes and CON.

### Gray matter density-based classifier performance across conventional diagnoses

**Figure 2:**
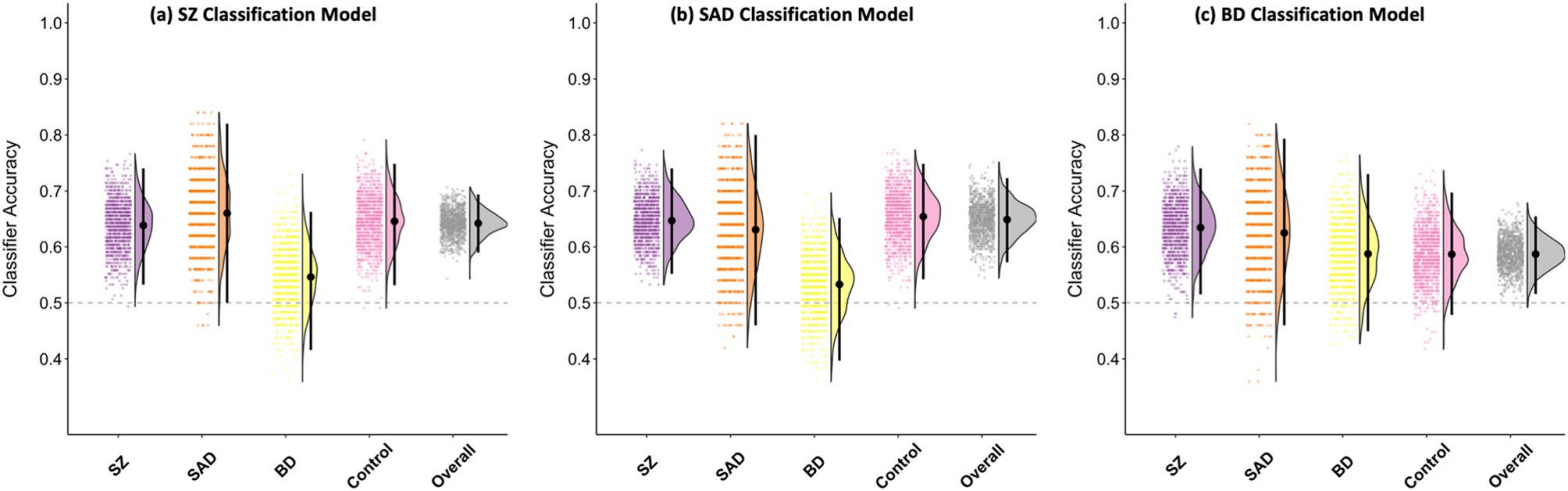
Raincloud plots from the analysis of the GMD classifiers for (**a**) Schizophrenia (SZ) versus Control (CON), (**b**) Schizoaffective disorder (SAD) versus CON, and (**c**) Bipolar disorder (BD) versus CON. In each panel, the dots represent balanced classifier accuracy for each of the 1000 iterations, the density plot shows the distribution of accuracy values across iterations, and the black dot and line reflects the mean and 99.17% interval of the accuracy values, respectively. Overall accuracy reflects on the two groups in the model (e.g., overall accuracy for the SZ model is the combined accuracy of SZ and CON cases). The other columns of the figure reflect accuracy for individual groups. Note that accuracy for groups not included in the training model (e.g., SZ and BD for the SZ model), the ‘accuracy’ value reflects the rate of classifier guesses for being in the psychosis group (e.g., SZ).

The results for the three conventional diagnoses classification models are shown in Fig. 2 and Supplemental Table S4. Overall classification accuracy for the SZ vs. CON model, as well as accuracies for both SZ and CON, were significantly above chance. However, the model misclassified SAD cases as belonging to SZ at above chance levels (at a similar rate to SZ cases). BD cases were not misclassified as SZ above nominal chance. Therefore, the SZ vs. CON model appeared to be nonspecific and driven by GMD features common to both SZ and SAD.

Overall classification accuracy for the SAD vs. CON model was significantly above chance. CON, but not SAD, group was classified at above chance rates. The model misclassified SZ cases as belonging to SAD at above chance level, while BD were not misclassified as SAD. Thus, similar to the SZ vs. CON model, the SAD vs. CON model appeared to be nonspecific and driven by brain structural characteristics common to SZ and SAD.

Lastly, overall classification accuracy for the BD vs. CON model was significantly above chance. However, neither classification accuracy for BD nor CON were above nominal chance. The model also misclassified SZ, but not SAD, as belonging to BD group at an above chance rate. Thus, classification performance of the BD vs. CON model appeared to be driven by GMD features common to BD and SZ, and CON.

### Feature weights for the classification models

The voxel-wise feature maps across the Biotype and conventional diagnosis groups (Supplementary Figs. S1 and S2) paralleled the spatial voxel-wise GMD maps from the original VBM analyses^5^. A stepwise distribution of classifier feature weights was observed across the Biotypes, with the most diffusely distributed, consistent and strong features in B1, considerably fewer consistently strong features in B3, and intermediate number of features in B2 (Supplemental Fig. S1). In contrast, across the conventional diagnoses (Supplemental Fig. S2), the consistent and strong classifier features were more spatially similar to each other, echoing our VBM findings of poor separation of the SZ, SAD and BD groups based on GMD. It is important to note that it is difficult to make claims as to whether the identified features are significant in a statistical sense, or if they are the most important. The contribution of a feature in any machine learning model in part, depends on the other features that are included in the model. Thus, these feature maps and results should not be taken as evidence that these features in isolation would produce the same results reported above in “Gray matter density-based classifier performance across the biotypes” and “Gray matter density-based classifier performance across conventional diagnoses”.

### Associations between gray matter density-based classifier performance for biotype 1 vs. controls and biomarker and clinical measures

We next explored whether classifier evidence indicating membership of the B1 group (derived from the B1 model which was the only model to show specificity) was associated with biomarker and clinical measures that were not used in the Biotype development, and if any of these relationships predicted performance regardless of group membership. Table 2 shows the results for all six measures. B1 classifier evidence demonstrated a significant negative association with a single measure, i.e., an estimate of a premorbid general intellectual ability (WRAT-4/Reading Subtest). Importantly, this relationship appeared to be group invariant: there was no evidence of a significant interaction between any group and B1 classifier evidence for WRAT-4 scores. No other significant associations were found.

**Table 2:** Summary of regression model results. Measures reflect the average beta coefficient across the 1000 iterations with the 95% confidence bounds in parentheses (Lower, Upper).

Outcome variable	Intercept	B1 class. evidence	B1 group	B1 class. by group interaction	B2 group	B2 class. by group interaction	B3 group	B3 class. by group interaction
WRAT-4/reading subtest	0.26* (0.15; 0.37)	− 0.42* (− 0.73; − 0.07)	− 1.25* (− 1.49; − 0.99)	0.58 (− 0.16; 1.26)	− 0.08 (− 0.26; 0.11)	0.28 (− 0.20; 0.75)	− 0.30 (− 0.45; − 0.15)	0.27 (− 0.16; 0.71)
GAF	158.15* (156.07; 160.18)	2.91 (− 3.22; 8.58)	− 40.09* (− 45.89; − 33.96)	− 6.03 (− 20.72; 9.66)	− 33.43* (− 37.55; − 29.37)	− 4.84 (− 16.72; 7.57)	− 26.04* (− 29.70; − 22.24)	− 10.72 (− 21.04; 0.52)
SFS	0.20* (0.09; 0.30)	− 0.32 (− 0.64; 0.01)	− 1.16* (− 1.38; − 0.95)	0.40 (− 0.18; 0.96)	0.01 (− 0.17; 0.19)	0.30 (− 0.24; 0.82)	− 0.31 (− 0.47; − 0.15)	0.09 (− 0.39; 0.56)
Intrinsic EEG PCA component 1	0.01 (-0.13; 0.15)	0.04 (− 0.41; 0.40)	0.06 (− 0.24; 0.36)	− 0.20 (− 0.94; 0.62)	− 0.03 (− 0.28; 0.24)	0.04 (− 0.61; 0.77)	0.01 (− 0.16; 0.20)	− 0.04 (− 0.55; 0.51)
SPEM PCA component 1	0.33* (0.24; 0.42)	− 0.04 (− 0.33; 0.24)	− 1.08* (− 1.39; − 0.78)	− 0.08 (− 0.86; 0.75)	− 0.67* (− 0.86; − 0.47)	− 0.00 (− 0.52; 0.54)	− 0.24* (− 0.37; − 0.11)	− 0.06 (− 0.49; 0.36)
SPEM PCA component 2	0.10 (− 0.03; 0.23)	0.28 (− 0.13; 0.70)	− 0.10 (− 0.46; 0.32)	− 0.38 (− 1.48; 0.61)	− 0.26* (− 0.45; − 0.05)	− 0.00 (− 0.64; 0.56)	− 0.09 (− 0.28; 0.09)	− 0.22 (− 0.75; 0.34)

*B1* Biotype 1, *B2* Biotype 2, *B3* Biotype 3, *WRAT-4* wide range achievement Test-4 (Reading Subtest), *GAF* the global assessment of functioning, *SFS* the birchwood social functioning scale (total score), *PCA* principal component analysis, *SPEM* smooth pursuit eye movement.

Asterisks (*) indicate regression parameters for which the 95% interval of the 1000 bootstrapped iterations did not include 0. The WRAT-4/Reading Subtest outcome variable is in bold font as it was the only variable to show a significant association with B1 classifier evidence (that was similar across all three groups).

## Discussion

Expanding on our prior work—the development of distinct B-SNIP psychosis Biotypes derived from cognitive and neurophysiologic biomarkers^3^ and the characterization of brain structural and functional alterations across Biotypes using independent neuroimaging measures^5,6^—here, we examined whether a supervised machine learning approach applied to voxel-wise GMD measures would successfully classify the Biotype and conventional diagnosis groups. Compared to symptom-based diagnoses, Biotypes appear to capture neurobiologically-distinctive and more homogeneous psychosis subgroups^3^. Therefore, we predicted that a GMD-based classifier would demonstrate more specificity for Biotypes than diagnoses. We also examined whether GMD-based classifier evidence was associated with several biomarker and clinical measures not used in Biotype definition.

Our results converge with and extend previous work using machine learning approaches to classify psychotic disorders using features derived from structural MRI^7–14,20–22^. First, we replicate previous findings indicating that patterns of GMD discriminate SZ and BD from CON at above chance rates. We also extend prior work by demonstrating that GMD can discriminate SAD [which is typically either not included into such analyses (e.g. Refs.^7,10,15^) or is merged with SZ cases (e.g. Ref.^11^)] from CON.

A novel extension of our study is the application of machine learning approaches to the discrimination of Biotypes—experimental neurobiologically-based categories of psychosis^3^. Notably, the classifier models were trained on whole-brain GMD features that were not used to derive Biotypes. All three Biotype models classified cases at above chance rates, indicating that patterns of GMD can discriminate between biologically-derived subgroups of psychosis (relative to CON). An interesting aspect of these findings is that classification accuracy demonstrated a gradient, such that it was numerically highest for the B1 model, intermediate for the B2 model, and lowest for the B3 model. This echoes both the overall pattern of GMD reductions relative to CON^5^ and the differing levels of cognitive impairment^3^ previously demonstrated across the three Biotypes.

One aim of the present study was to determine if machine learning models can capture features that are *specific* to categories of psychotic disorders. The inclusion of multiple psychosis subgroups allowed us to assess the specificity for both the Biotype- and conventional diagnosis-based classification schemes. We applied each of the models trained to classify one of the groups (e.g. B1 vs. CON) to the other two groups within the same classification scheme (e.g. B2 and B3), which allowed us to determine the rate at which the two groups not included in the model (e.g. B2 and B3) were classified as members of the psychosis group used to train the model (e.g. B1). The logic of this approach is that a model capturing GMD features specific to a particular psychosis subgroup would not lead to above chance “misclassification” of belonging to a different subgroup. Only the B1 model showed evidence of model specificity. That is, the B1 model classified only members of the B1 group, and not the B2 or B3 groups, as belonging to the B1 group at rates above nominal chance. Neither the B2 nor B3, nor any of the conventional diagnosis (see also Ref.^42^), models showed similar evidence of specificity. The lack of model specificity might explain the consistently modest classification rates observed when attempting differentiate SZ and BD in prior research^23^.

We also explored if classifier performance predicted individual differences in biomarker and clinical measures that were not used in Biotype creation. This analysis was restricted to classifier evidence from B1 vs. CON because it was the only model that demonstrated evidence of specificity. We aimed to explore whether meaningful relationships exist between the brain structure-based classifier evidence and a series of clinical and biomarker measures that could elucidate neurobiology/behavior interactions specific to individuals expressing B1-like patterns of GMD. Importantly, we chose analytic strategies that would allow capture of *dimensional* aspects of such relationships, independent of group membership or, indeed, of the distinction between psychosis cases and CON. We found that, among the six tested measures spanning cognition, EEG, ocular-motor, and general and social functioning, a single measure—an estimate of a premorbid general intellectual ability (WRAT-4/Reading Subtest)—demonstrated a negative association with GMD-based B1 classifier evidence. That is, a higher probability of being classified as B1, based on GMD characteristics, was associated with lower estimate of general intellectual ability. Importantly, we did not detect a significant interaction between any psychosis group and B1 classifier evidence for this measure, indicating that the relationship was group-invariant.

Single-word reading as assessed by the WRAT-4/Reading Subtest^43^ provides a widely used estimate of premorbid intellectual ability (in disease samples)^44^. It is considered to capture crystalized intellectual ability rather than the fluid/dynamic intellectual functions. While fluid cognitive function [as captured by the Brief Assessment of Cognition in Schizophrenia (BACS)] was used to discriminate the Biotypes^3^, and there is a relation between measures of fluid and crystalized intellectual ability, the correlations between BACS total scores and WRAT-4/Reading Subtest scores in our sample were moderate with only 10–15% shared variance (B1, r = 0.33, R^2^ = 0.11; B2, r = 0.31, R^2^ = 0.10; B3, r = 0.39, R^2^ = 0.15; all p < 0.05). Thus, measures of these two aspects of cognition each provide important and largely non-overlapping information in psychosis samples. The difference between aspects of cognition captured by WRAT and BACS has been previously used to assess psychosis-related cognitive decline in the B-SNIP sample^45^. Other reports from our group examined relationships between premorbid intellectual ability/WRAT-4 Reading measure and an array of biomarkers, including structural brain metrics^46^ and Polygenetic Risk for Schizophrenia^47^.

The association observed here between premorbid intellectual function and GMD-based probability of being classified as psychosis B1—regardless of group membership (psychosis or CON)—highlights the importance of the relationship between premorbid cognitive development and brain structure. Notably, WRAT-4 was the only measure that showed a significant association with the GMD-based Biotype classification. One important avenue for future research is to examine cognition/brain structure relationships over the course of development with the aim of identifying ‘high risk’ subgroups who may merit clinical monitoring. Another possibility is to explore cognition/brain structure interactions in prodromal and early psychosis samples, to test whether these features predict psychosis progression and broader functional outcomes. Further detailed investigation of relationships between cognition, brain structure and other biomarkers within and across psychosis Biotypes, and the replication of these relationships in independent samples, are essential to validate and extend our findings.

There are some limitations of the study that warrant mention. First, our classifiers were trained only with features derived from a single imaging modality. Anecdotally, previous research using features derived from multiple modalities to classify conventional diagnostic categories led to higher classification accuracy relative to studies using only a single imaging modality^16–19^. Using features from a single imaging modality might have underestimated classification accuracy and positively (or negatively) affected the results related to model specificity. Second, we did not directly compare the GMD-based classification outcomes between the Biotype vs. diagnosis categorizations. This requires further work and development in optimizing machine learning approaches to directly compare different classification schemes. Third, our findings require validation in independent samples suitable for “biotyping” based on a broad set of biomarker measures.

Together, our findings indicate that brain-based biomarker classification schemes, such as Biotypes, may hold promise in capturing disease features that are more specific to underlying psychosis neurobiology than are phenomenologically-defined diagnostic categories of psychosis. It is important to note that our results do not support the notion that the Biotype GMD-based classification scheme is superior (i.e., leads to higher accuracy) to the classifier performance possible for conventional diagnoses. Our primary conclusion is that Biotypes appear to be associated with more specificity in brain structure-based classification. This conclusion is based on the B1 model correctly classifying B1, but not B2 and B3 cases, at above chance rates. In contrast, none of the diagnosis classifier models demonstrated features of specificity, based on brain structure features. Future research is needed to further investigate the accuracy and model specificity associated with classification of psychosis based on neurobiological features.

### Supplementary Information

Supplementary Information.

## Data Availability

The B-SNIP dataset analyzed during the current study is available in the NIH Data Archive repository (https://nda.nig.gov). The data derivatives specific to the analyses reported here are available on the Open Science Framework (https://osf.io/9ra6j/?view_only=02b0bd7639c64bddbb6ecc4903c1e5d7).
