# Supplementary material for: Supervised machine learning classification of psychosis biotypes based on brain structure: findings from the Bipolar-Schizophrenia network for intermediate phenotypes (B-SNIP)
Source: Sci Rep. 2023 Aug 10;13:12980. doi: 10.1038/s41598-023-38101-0 (PMC10415369; doi:10.1038/s41598-023-38101-0)
Supplement: Supplementary file 1 — Supplementary Information. [file 41598_2023_38101_MOESM1_ESM.docx]

**Supplemental Information**

**Supplemental Methods**

**MRI-Pertinent Study Exclusion Criteria**

Subjects with organic brain disorders, history of brain insults accompanied by a loss of consciousness ≥ 30min, decompensated medical conditions, or substance abuse within 1 months or dependence within 3 months of enrollment were excluded; the drug-free status was confirmed by urine toxicology screen. Pregnant women, subjects with claustrophobia, those with irremovable ferromagnetic medical and non-medical objects lodged in body were also excluded.

**Site-Specific Imaging Acquisition Parameters**

All site followed the Alzheimer's Disease Neuroimaging Initiative (ADNI1) protocol (see <http://adni.loni.usc.edu/methods/documents/mri-protocols/> for full list of parameters). The MPRAGE or IR-SPGR parameters, as appropriate for each scanner brand/model, were as follows:

Site1, GE Signa (University of Chicago, Chicago IL): 3D acquisitions, sagittal slab, shot interval 2300ms, inversion time 700ms, TR 6.99ms, TE 2.85ms, flip angle 8º, FOV 260 (foot-to-head) x 260 (anterior-to-posterior) mm^2^, matrix 256x256, in-plane resolution 1x1 mm^2^, 166 slices, slice thickness 1.2 mm, voxel size 1x1x1.2mm^3^, total scan duration 10min 28sec.

Site2, Philips Achieva (UT Southwestern Medical Center, Dallas TX): 3D acquisitions, sagittal slab, shot interval 3000ms, inversion time 846ms, TR 6.8ms, TE 3.1ms, flip angle 8º, FOV 256 (foot-to-head) x 240 (anterior-to-posterior) mm^2^, matrix 256x240, in-plane resolution 1x1 mm^2^, 170 slices, slice thickness 1.2 mm, voxel size 1x1x1.2mm^3^, total scan duration 9min 19sec.

Site 3, Siemens Allegra (Olin Institute of Living, Hartford Hospital, Hartford CT): 3D acquisitions, sagittal slab, shot interval 2300ms, inversion time 900ms, TR 7.2ms, TE 2.91ms, flip angle 9º, FOV 256 (foot-to-head) x 240 (anterior-to-posterior) mm^2^, matrix 256x240, in-pane resolution 1x1 mm^2^, 160 slices, slice thickness 1.2mm, voxel size 1x1x1.2mm^3^, total scan duration 9min 14sec.

Site 4, Siemens Trio (Maryland Psychiatric Research Center, University of Maryland, Baltimore MD): 3D acquisitions, sagittal slab, shot interval 2300ms, inversion time 900ms, TR 6.8ms, TE 2.91ms, flip angle 9º, FOV 256 (foot-to-head) x 240 (anterior-to-posterior) mm^2^, matrix 256x240, in-pane resolution 1x1 mm^2^, 160 slices, slice thickness 1.2 mm, voxel size 1x1x1.2mm^3^, total scan duration 9min 14sec.

Site 5a: GE Signa HDxt (Harvard Medical School, Boston MA): 3D acquisitions, sagittal slab, inversion time 650ms, TR 7.0ms, TE 3.0ms, flip angle 8º, FOV 256 (foot-to-head) x 256 (anterior-to-posterior) mm^2^, matrix 256x256, in-pane resolution 1x1 mm^2^, 166 slices, slice thickness 1.2 mm, voxel size 1x1x1.2mm^3^, total scan duration 9min 58sec.

Site 5b: Siemens Trio (Wayne State University, Detroit MI): 3D acquisitions, sagittal slab, shot interval 2300ms, inversion time 900ms, TR 6.8ms, TE 2.74ms, flip angle 8º, FOV 176 (foot-to-head) x 256 (anterior-to-posterior) mm^2^, matrix 176x256x176, in-pane resolution 1x1 mm^2^, slice thickness 1.2mm, voxel size 1x1x1.2mm^3^, total scan duration 10min 09sec.

**Imaging Quality Control (QC) Procedures**

To ensure between- and within-site data quality and compatibility, standardized ADNI phantoms at each site were imaged weekly; drift in linear gradient calibration was monitored regularly throughout the duration of the study. Measurements of non-linear geometric fidelity and correction for gradient non-linearity occurred once (at the beginning of the study) at each site, and again in the event of a hardware upgrade. All T1 images were visually inspected by experienced imaging analysts before and at each step of image preprocessing, and scans were discarded if they did not meet QC standards. The initial imaging sample included 1,858 T1 scans. After QC procedures, a total of 1,681 scans were deemed usable for imaging analyses. One hundred and seventy seven scans were discarded based on either poor image quality (n=15, e.g., acquisition artifacts) or other excluding factors (n=162, e.g., active drug use detected after a subject already competed imaging session, a pilot subsample of non-psychotic bipolar probands and their relatives, etc.). Out of 1,681 usable scans, 1,409 were included in this analysis comprised of subjects who (i) were able to be categorized into Biotypes based on cognitive, oculo-motor and electrophysiological biomarker battery completion (see (1) for details) and (ii) had good-quality T1 structural scans.

**Voxel-Based Morphometry Analyses**

Whole brain voxel-based morphometry (VBM) (2) with the Diffeomorphic Anatomical Registration Through Exponentiated Lie Algebra (DARTEL) (3) was used to examine global and regional GMD biomarkers, and followed the standard steps in MATLAB2013a/SPM8/VBM8/DARTEL (3;4): (i) individual T1 images were manually reoriented to anterior-posterior commissure and segmented into gray matter (GM), white matter (WM), and cerebrospinal fluid components via the standard SPM8 segmentation algorithm; (ii) rigid body transformation parameters were extracted from the nonlinear deformations estimated by the segmentation algorithm and subsequently used to write out rigidly transformed versions of the tissue class images for each subject via DARTEL; (iii) the mean of the study images serving as a study-specific template was created and subsequently refined via the cyclic DARTEL iterations to ensure accurate registration while conserving each subject’s anatomical features informative for registering over subjects; (iv) GM and WM intensity averages were generated from the template, and nonlinear warping of the subjects’ GM and WM images was performed simultaneously to the tissue intensity averages; (v) warped GM images were used in the modulation step to ensure preservation of absolute amount of GM tissue corrected for individual brain size (5); the resulting voxel size in spatially normalized modulated images was 1.5x1.5x1.5mm^3^; (vi) modulated normalized GM images were smoothed with an 8-mm full width at half maximum (FWHM) and selected for group-level statistical analyses.

**Feature Importance Maps for Logistic Regression Classifiers**

The average feature weight for each voxel from the 1000 repeated train-test instances for each binomial classifier were computed to visualize the regions where GMD had the strongest association with group membership. The importance maps are shown in Supplemental Figures S1 and S2 for the biotype and conventional diagnosis models, respectively. Given that the weights were low on average, the voxel weights were multiplied by 1000 for visualization purposes. The feature importance maps were thresholded to only show voxels who’s 90% interval did not encompass zero. The aim of this was to identify the strongest and most consistent (across iterations) features contributing to group classification for each model. This should not, and cannot, be taken as evidence that these features are ‘significant’ in a statistical sense.

**Supplemental Table *S1*. The distribution of conventional diagnoses across the Biotype groups**

| **Biotypes** | **DSM-IV-TR Diagnoses** |
| --- | --- |
| Biotype 1, n=150 | SZ, n=91 (60.67%) SAD, n=34 (22.67%) BD, n=25 (16.67%) |
| Biotype 2, n=185 | SZ, n=80 (43.24%) SAD, n=49 (25.49%) BD, n=56 (30.27%) |
| Biotype 3, n=222 | SZ, n=71 (31.98%) SAD, n=55 (24.78%) BD, n=96 (43.24%) |

*Note.* SZ – probands with schizophrenia, SAD – probands with schizoaffective disorder, BD – probands with psychotic bipolar I disorder

**Supplemental Table *S2*. Proportions of missing clinical and biomarker data used in the classifier association analyses**

| **n (%)** | **CON**  **(n=251)** | **B1**  **(n=150)** | **B2**  **(n=185)** | **B3**  **(n=222)** |
| --- | --- | --- | --- | --- |
| **WRAT-4** | 8 (3.19) | 3 (2.00) | 5 (2.70) | 6 (2.70) |
| **GAF** | 5 (1.99) | 2 (1.33) | 1 (0.54) | 1 (0.45) |
| **SFS** | 70 (27.89) | 30 (20.00) | 35 (18.92) | 48 (21.62) |
| **IEA–C1** | 52 (20.72) | 41 (27.33) | 50 (27.03) | 55 (24.78) |
| **SPEM–C1** | 44 (17.53) | 27 (18.00) | 26 (14.05) | 30 (13.51) |
| **SPEM–C2** | 44 (17.53) | 27 (18.00) | 26 (14.05) | 30 (13.51) |

WRAT-4 – the Wide Range Achievement Test-4, Word Reading subtest, GAF – DSM-IV Axis V: Global Assessment of Functioning Scale, SFS – the Birchwood Social Functioning Scale, IEA–C1 – intrinsic EEG activity, PCA component1, SPEM–C1 – the Smooth Pursuit Eye Movement task, PCA component 1, SPEM–C2 – the Smooth Pursuit Eye Movement task, PCA component 2

**Supplemental Table S*3*.** **Mean [with 99.17% intervals] classification performance across the 1,000 iterations for the three Biotype models.**

|  | **Classification Accuracy** | | | | |
| --- | --- | --- | --- | --- | --- |
| **Model** | **Overall** | **B1** | **B2** | **B3** | **CON** |
| B1 vs. CON | .70 [.63; .76] | .69 [.54; .81] | *.57 [.45; .70]* | *.49 [.39; .60]* | .72 [.62; .80] |
| B2 vs. CON | .65 [.59; .71] | *.71 [.56; .84]* | .65 [.52; .78] | *.53 [.43; .63]* | .65 [.55; .75] |
| B3 vs. CON | .56 [.51; .61] | *.71 [.56 .85]* | *.65 [.49; .77]* | .57 [.45; .67] | .56 [.45; .67] |

*Note*. The values in brackets reflect the lower and upper bounds, respectively, of the 99.17% interval of the bootstrapped distribution derived from the 1,000 iterations. The underlined values in each row reflect the groups that contribute to the overall classification accuracy. Thus, these values reflect accurate classifications. The two values in italics for each model (row) reflect the proportion of cases in a group not included in model training being assigned to the patient proband. For instance, the B1 data for the B2 vs. CON model indicates that, on average across the 1,000 iterations, .71 of the B1 cases in the test set were classified as B2 cases (as compared to the CON group).

**Supplemental Table S*4*.** **Mean [with 99.17% intervals] classification performance across the 1,000 iterations for the three diagnosis models.**

|  | **Classification Accuracy** | | | | |
| --- | --- | --- | --- | --- | --- |
| **Model** | **Overall** | **SZ** | **SAD** | **BD** | **CON** |
| SZ vs. CON | .64 [.59; .69] | .64 [.53; .74] | *.66 [.50; .82]* | *.55 [.42; .66]* | .65 [.53; .75] |
| SAD vs. CON | .64 [.57; .72] | *.65 [.53; .74]* | .63 [.46; .80] | *.53 [.42; .66]* | .65 [.54; .75] |
| BD vs. CON | .59 [.51; .65] | *.63 [.52; .74]* | *.63 [.46; .79]* | .59 [.45; .73] | .59 [.48; .70] |

*Note*. The values in brackets reflect the lower and upper bounds, respectively, of the 99.17% interval of the bootstrapped distribution derived from the 1,000 iterations. The underlined values in each row reflect the groups that contribute to the overall classification accuracy. Thus, these values reflect accurate classifications. The two values in italics for each model (row) reflect the proportion of cases in a group not included in model training being assigned to the patient proband. For instance, the SZ data for the SAD vs. CON model indicates that, on average across the 1,000 iterations, .66 of the SZ cases in the test set were classified as SAD cases (as compared to the CON group).

**
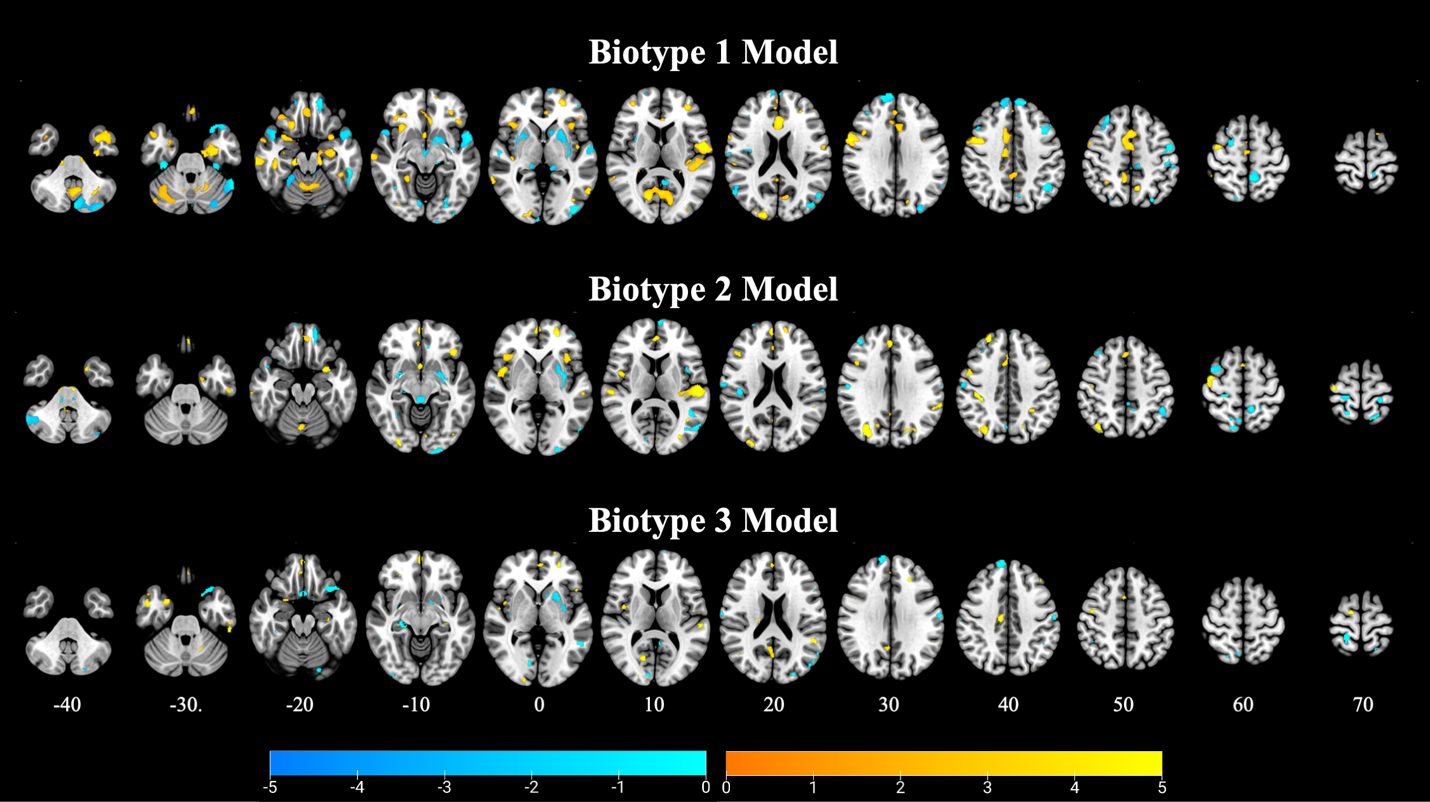
**

**Supplemental Figure *S1*.** Axial slices showing the average feature weights for the three Biotype binomial logistic classification models. Note that the weights are scaled by 1000 for visualization purposes. Thus, a weight of 4 equals a true weight of .004 in the model. The images are thresholded such that only voxels where the 90% interval of the feature weights across all 1000 model iterations did not encompass 0 (i.e., a *p* < .10 uncorrected threshold). This was done to identify the most consistent and strongest features across iterations and cannot be taken as evidence of ‘significance’.

**
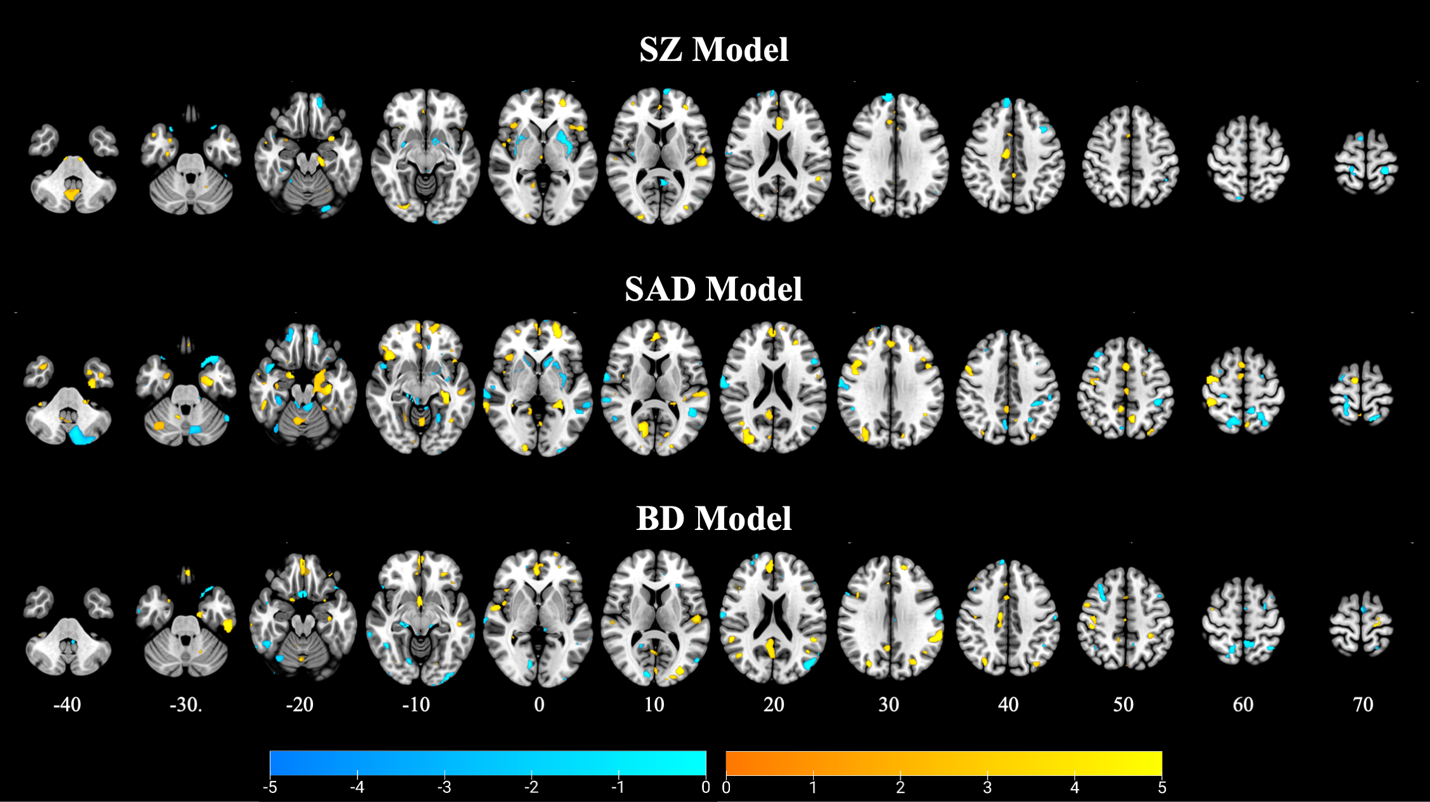
**

**Supplemental Figure S2.** Axial slices showing the average feature weights for the three conventional diagnosis binomial logistic classification models. Note that the weights are scaled by 1000 for visualization purposes. Thus, a weight of 4 equals a true weight of .004 in the model. The images are thresholded such that only voxels where the 90% interval of the feature weights across all 1000 model iterations did not encompass 0 (i.e., a *p* < .10 uncorrected threshold). This was done to identify the most consistent and strongest features across iterations and cannot be taken as evidence of ‘significance’.

**Supplemental References**

1. Clementz BA, Sweeney JA, Hamm JP, Ivleva EI, Ethridge LE, Pearlson GD et al. (2015): Identification of Distinct Psychosis Biotypes Using Brain-Based Biomarkers. *Am J Psychiatry* 173(4):373-384.

2. Ashburner J, Friston KJ. (2000): Voxel-based morphometry--the methods. *NeuroImage* 11(6 Pt 1):805-821.

3. Ashburner J. (2007): A fast diffeomorphic image registration algorithm. *Neuroimage* 38(1):95-113.

4. Kurth F, Luders, Gaser C (2010) VBM8-Toolbox Manual.

5. Good CD, Johnsrude IS, Ashburner J, Henson RN, Friston KJ, Frackowiak RS. (2001): A voxel-based morphometric study of ageing in 465 normal adult human brains. *Neuroimage* 14(1 Pt 1):21-36.
